# The influencing factors of college students’ legal emotion and the mechanism of its effect on aggressive behavior

**DOI:** 10.3389/fpsyg.2024.1295915

**Published:** 2024-04-18

**Authors:** Shuhui Xu, Junwen Yu, Lu Fan, Qingmei Yang, Zhiqiang Wang, Yuanyuan Zhang

**Affiliations:** ^1^Department of Psychology, School of Education, Wenzhou University, Wenzhou, China; ^2^Department of Mental Health Counseling Center, Xinyang College, Xinyang, China; ^3^Department of E-commerce, School of Business Management, Zibo Vocational Institute, Zibo, China

**Keywords:** legal emotion, aggressive behavior, emotional expression, adult attachment, social alienation

## Abstract

Current research has increasingly focused on the preventive role of individual legal socialization in crime. The socialization of legal emotions is an important part of legal socialization. Building upon existing literature, this study, conducted through two sub*-*studies, investigated the influencing factors of legal emotions in N mainland Chinese university students and the mechanisms through which legal emotions impact aggressive behavior. In study 1, the results indicated that mother*-*child attachment, innovation spirit, and positive emotional expression positively predicted positive legal emotion, while mother*-*child attachment, dependency dimension in adult attachment, and positive emotional expression negatively predicted negative legal emotions. The anxiety dimension in adult attachment and negative emotional expression positively predicted negative legal emotions. In study 2, Positive legal emotion among university students could directly negatively predict aggressive behavior or exert influence through social alienation. Negative legal emotions could not only directly positively predict aggressive behavior but also partly affect it through social alienation. In summary, our study not only identified factors that influence legal emotions, but also found that legal emotions have an impact on aggressive behavior directly or indirectly through social alienation. Our research findings have significant implications for cultivating positive legal emotion in university students and curbing aggressive behavior. This can be achieved by promoting the legal socialization of university students and ultimately contributing to crime prevention.

## 1 Introduction

### 1.1 The concept and dimensions of legal emotion

The theory of emotional social structure posits that emotional experiences are rooted in rational social contextual relationships, and emotions are acquired within the social and cultural system (Fu, 2016). Legal emotions constitute one of the categories within the process of emotional socialization. The existing legal system is, in fact, a recognition and commitment to the prevailing mainstream culture of society. During a certain period of societal development, when individuals collectively share a particular legal system, their cognitive evaluations of this legal system form the basis of legal emotions. These evaluations, rooted in the perception of the existing legal framework, are susceptible to the influence of others and simultaneously reflect the dynamics of interpersonal relationships. In other words, legal emotions are partly shaped through interpersonal interactions and can have an impact on interpersonal relationships. The manifestation of the social control power of legal emotions lies in their formation within interpersonal relationships and their subsequent role in shaping these relationships. Therefore, the developmental process of legal emotions is a crucial component of individual legal socialization.

Legal emotion is a subordinate concept of emotion. It is a kind of psychological phenomenon that reflects the individual’s desire and need, as well as the individual’s complex reaction to the spirit of the rule of law, the current legal system, and the related legal stimuli. Based on the concept of legal emotions, several characteristics can be inferred:

Firstly, legal emotions possess both individuality and collectivity. Legal emotions constitute a subjective experience of legal phenomena by individuals, reflecting the individual aspect of legal emotions, while also holding a collective nature. The collectivity is primarily evident in the following ways: In the first place, the substitutability of legal emotions, which means that legal events happening to other members can also trigger corresponding emotional responses in individuals. For instance, the feeling of pleasure that arises when seeing news reports about “wrongdoers” receiving severe legal penalties, or the sense of anger provoked by witnessing instances of legal injustice. Moreover, the collectivity is reflected in the similarity of legal emotions, where a majority of group members share analogous emotional experiences towards legal matters. For instance, affirming a sense of justice and feeling anger towards infringements are examples of these similar legal emotions. On one hand, these emotions stem from common human traits, such as the pursuit of truth, goodness, and beauty; on the other hand, they are influenced by shared moral concepts within the group. Morality, in turn, is a crucial source for the development of law. Once again, the social transmission aspect of legal emotions also demonstrates their collective nature, as legal emotions are transmitted among group members, thereby playing a regulatory role in shaping group attitudes and behaviors. For example, the collective anger or approval triggered by a particular court judgment. The social contagiousness of legal emotions also indicates the substitutability and shared character of legal emotions.

Secondly, the triggers of legal emotions stem from the spirit of the rule of law, the existing legal framework, and dynamic legislation. There are various types of individual emotions, and legal emotions specifically refer to the subjective experiences prompted by legal phenomena. Based on whether the object of emotional focus is abstract or concrete, these emotions can be categorized into two layers: The first layer involves emotions related to the spirit of the rule of law, such as the subjective experiences of justice, equality, democracy, freedom, and other principles embodied in the law; the second layer pertains to emotional states concerning the current legal framework. The current legal framework consists of two main components: the static and the dynamic. The static legal framework includes laws, administrative regulations, local regulations, and various legal departments under the guidance of the Constitution. The dynamic legal framework mainly includes processes such as legislation, law enforcement, and compliance. Legal emotions refer to the subjective experiences triggered by these legal phenomena.

Thirdly, the logical basis for the occurrence of legal emotions is the concept of justice, which embodies the spirit of the rule of law. When individuals violate rational agreements established to uphold mutual interests, legal emotions come into play. In this moment, the emotions arising within a legal context bear the hallmark of the law. At this point, these legal emotions are often categorized as individual legal emotions, as their emergence is rooted in personal interests being compromised and underpinned by the concept of reciprocal justice. Another form of justice is distributive justice, which points towards the concept of shared public good. When this form of justice is compromised, the legal system takes on the responsibility of administering distributive justice, either by making judgments or providing redress to the victims. During this transition, the legal emotions arising from the violation of justice become the underlying logic for the emergence of national legal sentiments. In other words, whether it’s individual legal emotions or national legal sentiments, the logical starting point for their emergence is the concept of justice, which embodies the spirit of the rule of law.

Fourthly, the occurrence of legal emotions is accompanied by a series of physiological arousal states. Legal emotions belong to the subordinate concept of emotions. What distinguishes emotions from other psychological processes is that their occurrence is always accompanied by physiological reactions and external manifestations. Similarly, legal emotions also possess these characteristics. In other words, when legal emotions occur, they trigger responses in an individual’s autonomic nervous system and somatic nervous system. This characteristic becomes the prerequisite for the measurement and quantification of legal emotions.

Based on the current research in the field of psychology regarding emotional structure, this study employs the Circular Model of Emotions theory, categorizing legal emotions into positive legal emotions and negative legal emotions based on their valence structure (Posner et al., 2005). Positive legal emotions refer to instances where the spirit of the rule of law or the current legal framework and the operation of laws meet individuals’ expectations or needs, resulting in a subjective experience of pleasure. Positive legal emotions convey an individual’s endorsement of the legal system, and these emotions are also an emotional state that a nation encourages and anticipates schools to cultivate through education. Negative legal emotions are characterized by an individual’s adverse emotional experience and sentiments towards the current legal system and its manifestations, reflecting a pessimistic evaluation of the law. In the field of crime prevention education, it is important to be vigilant about the causes and extent of negative legal emotions formation among adolescents.

### 1.2 The influencing factors of legal emotion

Parent*-*child attachment refers to a lasting and intense emotional bond between parents and their children (Feder and Diamond, 2016). Bronfenbrenner’s Ecological Systems Theory (1979) posits that the family serves as the starting point for individual development, and parent*-*child relationships play a crucial role in shaping an individual’s socialization (Buist et al., 2017). Individuals with poor parent*-*child relationships lack parental care and understanding, leading to the development of insecure attachment relationships with their parents. These insecure attachment patterns are internalized as internal working models for their interpersonal interactions (Riggs, 2010). Developmental psychologist Bowlby (1980, 1982) believed that parent-child attachment serves as a bridge for the emotional socialization of children. Children’s development of positive emotions relies on the extent to which caregivers satisfy their comfort and needs. Parent*-*child attachment, being a strong and enduring emotional bond between an individual and their caregivers, can influence the child’s later relationships with other social entities, such as peers, romantic partners, and even unfamiliar individuals.

Simultaneously, a positive parent*-*child attachment also lays the foundation for the healthy development of personality. As an advanced form of socialized emotions, legal emotions emerge within the context of an individual’s emotional socialization process. The development of legal emotions, as a crucial component of legal socialization, is intertwined with factors that influence individual legal socialization. These factors are equally significant in shaping the development of individual legal emotions. Relevant studies indicate that the process of individual legal socialization is influenced by people in their surroundings, such as peers, neighbors, and family members (Fagan and Tyler, 2005). The formation and development of legal socialization are directly influenced by parents (Cavanagh et al., 2022). This is because the development of legal*-*related beliefs starts during childhood, as parents instill notions of right and wrong and guide children’s positive attitudes toward authority figures (Wolfe et al., 2016). A longitudinal study showed that the quality of intimate relationships, partner supervision, and partners’ prosocial orientation all predicted an increase in their perception of legal legitimacy and a decrease in their cynicism about legal norms (Forrest, 2021). Based on this, the current study proposes two crucial influencing factors for the formation of legal emotions: parent*-*child attachment and adult attachment.

According to the relevant view of creativity and emotion, the difference in the mechanism of threat on creativity is due to the influence of different threat levels, differences in creativity mechanism, and related mediating variables (Yi et al., 2021). An individual’s creativity is also influenced by their sense of psychological safety (Dong and Wang, 2021). Individuals exhibit significantly higher creativity in a positive emotional state compared to a negative emotional state (Lu et al., 2002). As an advanced form of socialized emotion, does legal emotion also get influenced by an individual’s creativity? Emotional expression is a personality trait of individuals, which refers to the process of expressing their emotions through explicit behavior. Relevant research indicates that emotional expression can influence feelings of loneliness in left*-*behind children (Li et al., 2021). Emotional expression, as an emotion regulation strategy, acts as a protective factor by moderating the relationship between childhood trauma and negative automatic thinking (Yu et al., 2021). Hence, what mechanism does emotional expression have in shaping legal emotions? Does it exhibit differential effects on positive legal emotions versus negative legal emotions? In light of this, the current study takes an individual*-*centered approach and suggests two other variables that could potentially influence legal emotions: innovative mindset and emotional expression.

### 1.3 Legal emotions and aggressive behavior

Studies have shown that adolescents with a negative attitude toward the law are more likely to be violent (Nivette et al., 2020). Legal cynicism has also become an increasing focal point for understanding the connection between individuals’ attitudes toward the judicial system and their engagement in criminal behavior (Gifford and Reisig, 2019). Legal cynicism reflects a dismissive and pessimistic attitude towards the law (Sampson and Bartusch, 1998). Specifically, individuals exhibiting legal cynicism do not accept social norms that serve as the foundation of the law, leading them to believe that “laws or rules are not perceived as binding.” Individuals’ cynicism towards the law might stem from past experiences of unfair treatment during arrests or from disappointment over insufficient punishment for crimes. This could eventually lead them to view law*-*breaking as reasonable (Sampson and Bartusch, 1998). The concept of legal cynicism here closely aligns with the negative legal emotions as defined in this study. Adolescents who hold a skeptical attitude toward the judicial system tend to be more susceptible to criminal behavior (Fagan and Tyler, 2005). Individuals who exhibit legal cynicism and those who deny the legitimacy of the criminal justice system and its agents are also more likely to engage in criminal behavior (Kaiser and Reisig, 2019). Conversely, individuals with higher scores in perceiving legitimacy are less likely to engage in criminal activities (Tankebe et al., 2016). Tyler initially believed that legitimacy is citizens’ trust in the judicial system and the protection of people’s rights and interests by police, courts, and other relevant personnel in the judicial process (Tyler et al., 2014). This sense of trust in the judicial system falls under the category of positive legal emotions as defined in this study. The core of legal socialization is to legitimize legal authorities such as the police and the courts (Fagan and Tyler, 2005). Legal authority behavior is perceived as just, leading individuals to be more inclined to internalize prosocial norms (Tyler, 2006). Legal socialization is the process in an individual’s development that shapes their orientation, beliefs, and attitudes toward the legal system (Fagan and Tyler, 2005). Legal emotions form a crucial part of legal socialization. Negative legal emotions are linked to engagement in criminal behavior, while positive legal emotions are associated with prosocial behavior.

Relevant study has shown that there is a strong correlation between early aggression and later violent crime (Farrington, 1994). Aggression can significantly predict criminal behavior (Ang et al., 2016). Adolescents with higher levels of aggression are more prone to engaging in criminal activities (Kabasakal and Baş, 2010). Early aggressive behavior in individuals can predict later criminal behavior (Loeber and Dishion, 1983). Therefore, the second research theme of this study focuses on the relationship between legal emotions and aggressive behavior.

According to Social Control Theory, maintaining social stability and order can be achieved by shaping individuals’ moral values and beliefs, as well as by utilizing societal norms such as laws (Hirschi, 2002). Social Capital Theory posits that social networks hold value for both individuals and society, as individuals obtain information, support, and trust from these networks to maintain societal stability (Bourdieu, 1986). Based on this theory, the second part of this study introduces the concept of social alienation to assess the relationship between individuals and society. Social alienation refers to a negative emotional experience arising from individuals being disconnected from society, and lacking social support, or meaningful social connections (Mau, 1992). Therefore, can legal emotions influence individual aggressive behavior through social alienation?

The General Aggression Model (GAM) offers an explanation for aggressive behavior. According to this theory, individual factors (such as traits and attitudes) and situational factors, along with their interactions, impact an individual’s cognition, emotions, physiological arousal, and ultimately lead to the activation of aggressive behavior (Anderson and Bushman, 2002). Dodge argued that aggressive behavior manifests as a lack of social skills and serves as an indicator of deteriorating social relationships (Crick and Dodge, 1994). At the same time, the social cognitive information processing model, which explains the causes of individual aggressive behavior, holds that emotional factors affect each step of individual information processing (Lemerise and Arsenio, 2000). Therefore, it is worth exploring the mechanisms through which legal emotions impact aggressive behavior, as well as the role of social alienation in this process.

### 1.4 The current study

As a significant component of legal socialization, fostering positive legal emotions among university students is crucial for public safety and crime prevention. This study draws on relevant literature in developmental psychology to examine the factors influencing the formation and development of legal emotions during the legal socialization process of university students, as well as the mechanisms through which these legal emotions affect aggressive behavior.

In specific terms, this study aims to address several questions through two sub*-*studies. Firstly, within the process of forming legal emotions among university students, what is the impact of various social relationships? How do father*-*son attachment, mother*-*son attachment, and adult attachment differ in their explanatory power regarding university students’ legal emotions? Secondly, in alignment with China’s emphasis on cultivating innovative qualities in university students as part of their quality education, this study intends to reveal the influence of innovation spirit and emotional expression on the development of legal emotions among university students. Lastly, are university students who hold negative attitudes towards the law more prone to exhibiting aggressive behavior? To what extent are they influenced by the social relationships of university students? Based on previous literature and relevant theories, we anticipate that social alienation plays a mediating role in this relationship.

## 2 Study 1: factors and mechanisms influencing legal emotions among college students participants

### 2.1 Study 1 method

#### 2.1.1 Sample

College students from a university in Wenzhou, Zhejiang Province, China, were selected as participants. The main experimenter was a psychology major student who had received professional training in administering tests. During a class session, the students were instructed by the trained psychology student to scan a QR code and participate in a group assessment. The initial collection yielded 507 valid responses. Out of these, 172 were from male students and 335 were from female students. The distribution across academic years was as follows: 52 freshmen, 67 sophomores, 128 juniors, and 260 seniors. Furthermore, there were 298 participants from humanities disciplines and 209 from science disciplines. In the second round of data collection, there were 323 valid responses. Among these, 103 were from male students and 220 were from female students. The distribution across academic years was as follows: 39 freshmen, 76 sophomores, 58 juniors, and 150 seniors. Additionally, there were 161 participants from humanities disciplines, 45 from medical disciplines, 133 from engineering disciplines, and 10 from art disciplines.

#### 2.1.2 Procedures

The questionnaire response time for this study was approximately 20–25 min. Each participant was provided with informed consent, and to protect the privacy of the participants, the survey was conducted anonymously. This research obtained approval from the Ethics Committee of Wenzhou University.

#### 2.1.3 Measures

*The college student legal emotion scale* was developed by the research team led by Xu (2020). The scale consists of two subscales: Positive Legal Emotions and Negative Legal Emotions. The scale consists of a total of 33 items, with the Positive Legal Emotions subscale containing 11 items and the Negative Legal Emotions subscale containing 22 items. The Likert 5*-*point rating scale was used for the assessment, where scores ranging from 1 to 5 represent responses from “Strongly Disagree” to “Strongly Agree.” Exactly, in this context, higher scores on the Positive Legal Emotions subscale indicate higher levels of positive legal emotions experienced by the participants. Similarly, higher scores on the Negative Legal Emotions subscale indicate higher levels of negative legal emotions experienced by the participants. The Cronbach’s α coefficients for the two subscales are 0.886 and 0.948, respectively. In this study, the Cronbach’s α coefficients for the Positive Legal Emotions subscale and the Negative Legal Emotions subscale of the scale were 0.911 and 0.970, respectively.

*The parent-child attachment questionnaire* employed the Father*-*Child Attachment and Mother*-*Child Attachment subscales of the Revised Inventory of Parent and Peer Attachment (IPPA*-*R) (Zhang et al., 2011). Each subscale comprises three dimensions: Trust, Communication, and Alienation, with 25 items in each subscale. A five*-*point Likert scale scoring method is employed, ranging from “Completely Agree” to “Completely Disagree.” Higher scores indicate a more negative experience of parent*-*child relationships for the individual. In this study, the Cronbach’s α coefficient for the Father*-*Child Attachment subscale was 0.909, and the Cronbach’s α coefficient for the Mother*-*Child Attachment subscale was 0.885.

*The revised adult attachment scale* (Collins, 1996) comprises 18 items, organized into three dimensions: Closeness, Anxiety, and Dependence. Each dimension consists of 6 items. The scale employs a five*-*point Likert scale scoring method, ranging from “Completely Agree” to “Completely Disagree.” In this study, the Cronbach’s α coefficients for the Closeness and Anxiety dimensions of the scale were both above 0.7, and the Cronbach’s α coefficient for the Dependence dimension was 0.62.

*The college students’ innovation spirit scale* was developed by Wang Hongli and his colleagues (Wang and Liu, 2009). The questionnaire includes two dimensions: Subject and Object, as well as seven sub*-*dimensions. It is used to assess the participants’ problem awareness, critical spirit, critical thinking abilities, problem identification and formulation skills, as well as their questioning attitude, and the spirit of exploring the unconventional and seeking novelty and innovation. The questionnaire consists of a total of 40 items and employs a five*-*point Likert scale scoring method, where scores range from 1 to 5, indicating responses from “Completely Disagree” to “Completely Agree.” The questionnaire’s Cronbach’s α coefficient is 0.902, and the test*-*retest reliability after a 10*-*day interval is 0.897. In this study, the questionnaire’s Cronbach’s α coefficient is 0.849.

*The emotional expression questionnaire* was developed by Wang Zhenhong and colleagues (Shen and Chen, 2014). The questionnaire is divided into two subscales: Positive Emotional Expression and Negative Emotional Expression. These two subscales are either weakly correlated or not correlated, and thus cannot be combined to form a total emotional expression score. Emotion expression can be considered a stable personality trait of individuals. The questionnaire consists of a total of 18 items, with odd*-*numbered items representing Positive Emotion Expression and even*-*numbered items representing Negative Emotion Expression. The questionnaire utilizes a five*-*point Likert scale scoring method, where scores ranging from 1 to 5 indicate responses from “Completely Disagree” to “Completely Agree.” In this study, the Cronbach’s α coefficients for the two separate subscales were 0.849 and 0.915, respectively.

#### 2.1.4 Data analysis

The data processing and analysis were performed using SPSS 23.0 and the SPSS macro program PROCESS developed by Hayes (2013). The statistical analysis methods primarily involved correlation analysis, regression analysis, and tests for mediation effects.

### 2.2 Study 1 results

#### 2.2.1 Common method bias test

After the normal distribution test, the data conforms to a normal distribution. The Harman’s single*-*factor test was employed to analyze common method bias in the survey data. For the first round of data collection, a factor analysis was conducted on the 101 items. The results revealed that there were 18 common factors with eigenvalues greater than 1, accounting for 67.039% of the variance. The variance explained by the first factor was 19.87%, which was below the critical threshold of 40%. This suggested that there was no significant presence of common method bias in the data collected during the first round. For the second round of data collection, a factor analysis was performed on the 94 items. The results indicated that there were 15 common factors with eigenvalues greater than 1, accounting for 69.947% of the variance. The variance explained by the first factor was 24.022%, which was below the critical threshold of 40%. This suggested that there was no significant presence of common method bias in the data collected during the second round.

#### 2.2.2 Correlation analysis between legal emotions and various influencing factors

Correlation between legal emotion and adult attachment, parent*-*child attachment. The results of the correlation analysis indicated that positive legal emotion was significantly positively correlated with parental attachment, adult attachment’s closeness, and dependence subscales, while it was significantly negatively correlated with the anxiety subscale of adult attachment. Negative legal emotion was significantly negatively correlated with parental attachment, closeness, and dependence subscales, and significantly positively correlated with the anxiety subscale of adult attachment. Please refer to the table for specific results. Specific results are shown in Table 1.

**Table 1:** Correlation analysis between legal emotions and adult attachment, parent-child attachment.

	*M ± SD*	Positive legal emotion	Negative legal emotion	Mother*-*Child attachment	Father*-*Child attachment	Closeness	Dependence	Anxiety
Positive legal emotion	20.659 ± 5.628	1						
Negative legal emotion	89.466 ± 19.158	*−*0.327^***^	1					
Mother*-*Child attachment	62.485 ± 14.273	0.222^***^	*−*0.268^***^	1				
Father*-*Child attachment	67.809 ± 16.458	0.191^***^	*−*0.155^***^	0.552^***^	1			
Closeness	16.610 ± 3.075	0.115^**^	*−*0.233^***^	0.320^***^	0.246^***^	1		
Dependence	17.341 ± 3.160	0.079	*−*0.346^***^	0.290^***^	0.166^***^	0.483^***^	1	
Anxiety	18.529 ± 4.062	*−*0.115^**^	0.345^***^	*−*0.287^***^	*−*0.232^***^	*−*0.315^***^	*−*0.379^***^	1

*p* > 0.05, * *p* < 0.05, ** *p* < 0.01, *** *p* < 0.001.

Relationship between legal emotion and innovative spirit, emotional expression among college students. The correlation analysis results revealed that positive legal emotion was significantly positively correlated with innovative spirit, positive emotional expression, and negative emotional expression. Negative legal emotion was significantly positively correlated with negative emotional expression, while its correlation with positive emotional expression was not statistically significant. Detailed findings can be found in Table 2.

**Table 2:** Correlation analysis between legal emotions and innovative spirit, emotional expression.

	M ± SD	Negative legal emotion	Positive legal emotion	Innovative spirit	Positive emotional expression	Negative emotional expression
Negative legal emotion	1.926 ± 1.104	1				
Positive legal emotion	4.212 ± 0.744	*−*0.185^***^	1			
Innovative spirit	128.085 ± 15.042	0.029	0.396^***^	1		
Positive emotional expression	3.746 ± 0.752	*−*0.009	0.437^***^	0.497^***^	1	
Negative emotional expression	3.270 ± 0.840	0.324^***^	0.170^**^	0.177^***^	0.516^***^	1

*p* > 0.05, *p < 0.05, **p < 0.01, *** *p* < 0.001.

#### 2.2.3 Regression analysis of various influencing factors on positive legal emotion

Regression analysis of adult attachment and parent*-*child attachment on positive legal emotion. The results of stepwise multiple regression analysis revealed that among the five predictor variables—Mother*-*children attachment, father attachment, and the three dimensions of adult attachment—only mother*-*children attachment exhibited significant predictive power. The multiple correlation coefficient between predictor variables and positive legal emotion was 0.222. The coefficient of determination, *R^2^*, was 0.049. The overall test of the regression model yielded an *F-*value of 26.207 (*p* = 0.000 < 0.05), indicating that the predictor variables together can effectively explain 4.9% of the variance in positive legal emotion. Looking at the standardized regression coefficients, the *β* values of predictor variables in the regression model are positive, indicating a positive influence on positive legal emotion.

Regression analysis of innovative spirit and emotional expression on positive legal emotion. The results of multiple regression analysis indicated that the correlation coefficient between the three independent variables and positive legal emotion was 0.485. The squared multiple correlation coefficient was 0.235, indicating that the three independent variables together could explain 23.5% of the variance in positive legal emotion. If the standardized regression coefficient of an independent variable was positive, it signified a positive influence on positive legal emotion. Conversely, if the standardized regression coefficient was negative, it indicated a negative influence on positive legal emotion. In the regression model, the predictor variables that significantly influenced positive legal emotion were innovative spirit and positive emotional expression. Among them, the absolute value of the standardized regression coefficient *β* for positive emotional expression was greater than that of innovative spirit. This indicated that positive emotional expression had a stronger explanatory power for positive legal emotion. The regression coefficient for negative emotional expression was not significant, suggesting that its explanatory power for the variance in positive legal emotion, as the dependent variable, was minimal, see Table 3 for details.

**Table 3:** Regression analysis of innovative spirit and emotional expression on positive legal emotion.

Variables	*B*	*SE*	Beta (*β*)	*t*
Constant	1.599	0.296		5.398^***^
Innovative spirit	0.011	0.003	0.231	4.476^***^
Positive emotional expression	0.345	0.059	0.348	5.860^***^
Negative emotional expression	*−*0.045	0.046	*−*0.05	*−*0.959
*R* = 0.485, *R^2^* = 0.235, △*R^2^* = 0.229, *F* = 39.381^***^				

*p* > 0.05, ^***^ *p* < 0.001.

#### 2.2.4 Regression analysis of various influencing factors on negative legal emotion

Regression analysis of adult attachment and parent*-*child attachment on negative legal emotion. The results of stepwise multiple regression analysis indicated that among the five predictor variables *-* the three dimensions of parental attachment and adult attachment *-* three had significant predictive power. These predictors were mother*-*children attachment, and the dependence and anxiety dimensions of adult attachment. The multiple correlation coefficient between the three predictor variables and negative legal emotion was 0.436. The coefficient of determination, *R^2^*, was 0.190. The overall test of the regression model yielded an *F-*value of 10.851 (*p* = 0.000 < 0.05), indicating that the three predictor variables together can effectively explain 19% of the variance in negative legal emotion. Among them, the variance explained by the dependence dimension of adult attachment was 11.9%, the variance explained by the anxiety dimension of adult attachment was 5.3%, and the variance explained by mother*-*children attachment was 1.7%. looking at the standardized regression coefficients, the *β* values of the three predictor variables in the regression model were *−*0.221, 0.221, and *−*0.141, respectively. A negative value indicates a negative influence on negative legal emotion, while a positive value indicates a positive influence, see Table 4 for details.

**Table 4:** Regression analysis of adult attachment and parent-child attachment on negative legal emotion.

Variables	*R*	*R^2^*	△*R^2^*	*F*	△*F*	*B*	*β*
Constant						105.239	
Dependence	0.346	0.119	0.119	68.493^***^	68.493^***^	*−*1.340	*−*0.221
Anxiety	0.416	0.173	0.053	52.666	32.558^***^	1.041	0.221
Mother-Child attachment	0.436	0.190	0.017	39.414^***^	10.851^***^	*−*0.189	*−*0.141

*p* > 0.05, ^***^ *p* < 0.001.

Regression analysis of innovative spirit and emotional expression on negative legal emotion. From the results of the multiple regression analysis shown in Table 5, it was observed that the correlation coefficient between the three independent variables and negative legal emotion was 0.392. The squared multiple correlation coefficient was 0.154, indicating that the three independent variables together could explain 15.4% of the variance in negative legal emotion. If the standardized regression coefficient of an independent variable was positive, it signified a positive influence on negative legal emotion. Conversely, if the standardized regression coefficient was negative, it indicated a negative influence on negative legal emotion. In the regression model, the predictor variables that significantly influenced negative legal emotion were negative emotional expression. Among them, the absolute value of the standardized regression coefficient *β* for negative emotional expression was greater than that of positive emotional expression. This indicated that negative emotional expression had a stronger explanatory power for negative legal emotion. The regression coefficient for innovative spirit was not significant, suggesting that its explanatory power for the variance in negative legal emotion, as the dependent variable, was minimal.

**Table 5:** Regression analysis of innovative spirit and emotional expression on negative legal emotion.

Variables	*B*	*SE*	Beta (*β*)	*t*
Constant	0.688	0.462		1.489
Innovative spirit	0.007	0.004	0.093	1.707
Positive emotional expression	*−*0.428	0.092	*−*0.291	*−*4.664^***^
Negative emotional expression	0.602	0.072	0.458	8.314^***^
*R* = 0.392, *R*^2^ = 0.154, △*R*^2^ = 0.147, *F* = 23.278^***^				

*p* > 0.05, *** *p* < 0.001.

#### 2.2.5 Mechanisms of various influencing factors on college students’ legal emotions

Building upon the results of the previous correlation and regression analyzes, we selected the influential factors with significantly high predictive power for legal emotions. We further aimed to uncover the internal mechanisms underlying their relationships.

Utilizing the Process macro developed by Hayes, we examined the impact of mother*-*children attachment on negative legal emotion among college students, specifically investigating the mediating roles of adult attachment anxiety and dependence. The results of sequential examinations are presented in Table 6. Mother*-*children attachment was found to significantly predict dependence and negative legal emotion. Additionally, both Mother*-*children attachment and dependence jointly predicted anxiety significantly. Moreover, Mother*-*children attachment, dependence, and anxiety collectively predicted negative legal emotion significantly. These findings suggest that in the context of the impact of mother*-*children attachment on negative legal emotion, the mediating roles of anxiety and dependence were significant.

**Table 6:** The mediation effect of anxiety and dependence on the association between mother*-*children attachment and negative legal emotion.

Variables	Negative legal emotion		Negative legal emotion		Anxiety		Dependence	
	*β*	*t*	*β*	*t*	*β*	*t*	*β*	*t*
Constant	84.009	9.023^***^	38.323	6.226^***^	30.362	20.688^***^	14.696	14.373^***^
Mother*-*Children attachment	*−*0.128	*−*3.037^**^	*−*0.254	*−*5.991^***^	*−*0.196	*−*4.687^***^	0.284	6.648^***^
Dependence	*−*0.198	*−*4.519^***^			*−*0.328	*−*7.816^***^		
Anxiety	0.240	5.435^***^						
Gender	0.168	4.167^***^	0.160	3.738^**^	*−*0.123	*−*3.030^**^	*−*0.075	*−*1.739
Grade	0.047	1.164	0.081	1.900	0.091	2.247^*^	*−*0.045	*−*1.053
Major	0.036	0.914	0.277	0.646	*−*0.038	*−*0.936	*−*0.001	*−*0.015
F	23.645^***^		14.783^***^		25.066^***^		12.791^***^	
R^2^	0.221		0.105		0.200		0.093	

*p* > 0.05, ^**^ *p* < 0.01, ^***^ *p* < 0.001.

**Figure 1:**
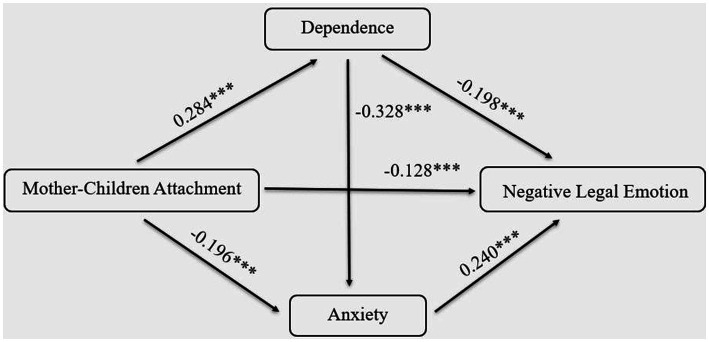
The Relationship between Mother-Child Attachment and Negative Legal Emotions: A Chain Mediation Model of Dependence and Anxiety.

The results of the direct test for the mediating effects are presented in Table 7. The results indicated the following: In the total indirect effects generated by the anxiety and dependence of adult attachment, the confidence interval between the upper and lower bounds of the bootstrap did not include the value 0, indicating a significant mediating effect between these two mediating variables in the relationship between mother*-*children attachment and negative legal emotion. Further analysis revealed that this mediating effect comprised three indirect effects: Indirect effect 1, mother*-*Children attachment → dependence → negative legal emotion, with a significant indirect effect; Indirect effect 2, Mother*-*children attachment → anxiety → negative legal emotion, also showing a significant indirect effect; Indirect effect 3, mother*-*children attachment → dependence → anxiety → negative legal emotion, with a significant indirect effect as well. The model is shown in Figure 1.

**Table 7:** Mediation effects with bootstrapping.

	Effect	BootSE	BootLLCI	BootULCI	Proportion
Indirect	0.169	0.033	0.109	0.24	0.496
Ind1	0.076	0.022	0.039	0.123	0.223
Ind2	0.063	0.019	0.031	0.102	0.185
Ind3	0.03	0.01	0.015	0.052	0.088
Direct	0.172	0.053	0.069	0.28	0.504
Total	0.341	0.049	0.249	0.443	

Ind1: Mother-Children attachment → dependence → negative legal emotion, Ind2: Mother-Children attachment → anxiety → negative legal emotion, Ind3: Mother-Children attachment → dependence → anxiety → negative legal emotion.

Using the Process macro program developed by Hayes, the relationship between innovative spirit and positive legal emotion among college students was analyzed with positive emotional expression as a mediating variable. Innovative spirit served as the independent variable, positive legal emotion as the dependent variable, and positive emotional expression as the mediating variable. Gender, grade, and major variables were controlled for. A multiple hierarchical regression analysis was conducted based on Model 4 in the Process program. The results of the analysis, as shown in Table 8, revealed the following: Innovative spirit significantly predicted positive legal emotion, innovative spirit significantly predicted positive emotional expression, and both innovative spirit and positive emotional expression significantly predicted positive legal emotion. Even after adding positive emotional expression, the impact of innovative spirit on positive legal emotion remained significant. Therefore, positive emotional expression partially mediated the relationship between innovative spirit and positive legal emotion.

**Table 8:** The mediation effect of positive emotional expression on the association between innovative spirit and positive legal emotion.

Variables	Positive legal emotion		Positive legal emotion		Positive emotional expression	
	*β*	*t*	*β*	*t*	*β*	*t*
Constant	1.841	5.545^***^	1.991	5.314^***^	0.313	*−*0.958
Innovative spirit	0.215	3.613^***^	0.389	7.518^***^	0.540	11.768^***^
Positive emotional expression	0.323	5.324^***^				
Gender	0.087	1.682	0.019	*−*0.362	*−*0.210	*−*4.558^***^
Grade	*−*0.037	*−*0.735	*−*0.043	*−*0.819	*−*0.018	*−*0.394
major	*−*0.022	*−*0.435	*−*0.002	*−*0.034	0.062	1.344
R^2^	0.226		0.157		0.338	
F	18.589^***^		14.876^***^		40.791^***^	

*p* > 0.05, *** *p* < 0.001.

**Figure 2:**
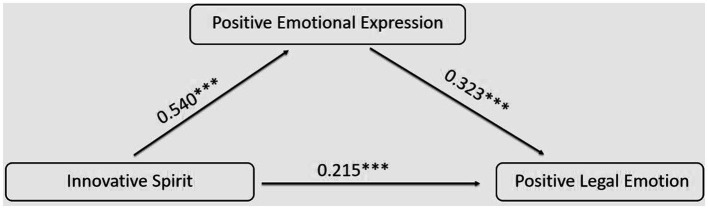
The Relationship between Innovative Spirit and Positive Legal Emotion: A Model Diagram of the Mediating Effect of Positive Emotional Expression.

Using the Bootstrap method with 5,000 resampling iterations, 95% confidence intervals were calculated for each tested path. The results revealed that the confidence intervals for all the paths examined did not include 0, indicating significant mediating effects. Among them, the direct effect accounted for 55.4%, while the indirect effect accounted for 44.6% of the total effect (Table 9). The model is shown in Figure 2.

**Table 9:** Mediation effects with bootstrapping.

	Effect	BootSE	BootLLCI	BootULCI	Proportion
Indirect	0.008	0.002	0.005	0.013	0.446
Direct	0.010	0.003	0.005	0.016	0.554
Total	0.019	0.003	0.013	0.024	

Using the Process macro program developed by Hayes, the relationship between college students’ negative emotional expression and negative legal emotion was analyzed with the mediating effect of positive emotional expression. Negative emotional expression served as the independent variable, negative legal emotion as the dependent variable, and positive emotional expression as the mediating variable. Gender, grade, and major variables were controlled. Based on Model 4 in the Process program, a multiple hierarchical regression analysis was conducted. The test results revealed (as shown in the table) that negative emotional expression significantly predicted negative legal emotion. Additionally, negative emotional expression significantly predicted positive emotional expression, and both negative and positive emotional expressions significantly predicted negative legal emotion. Even after accounting for positive emotional expression, the impact of negative emotional expression on negative legal emotion remained significant. Therefore, positive emotional expression partially mediated the relationship between negative emotional expression and negative legal emotion, see Table 10 for details.

**Table 10:** The mediation effect of positive emotional expression on the association between negative emotional expression and negative legal emotion.

Variables	Negative legal emotion		Negative legal emotion		Positive emotional expression	
	*β*	*t*	*β*	*t*	*β*	*t*
Constant	1.430	3.729^***^	1.769	4.680^***^	2.287	22.434^***^
Negative emotional expression	0.431	7.348^***^	0.333	6.463^***^	0.493	10.307^***^
Positive emotional expression	*−*0.198	*−*3.327^**^				
Gender	0.182	3.522^**^	0.214	*−*4.167^***^	*−*0.165	3.459^**^
Grade	*−*0.092	*−*1.810	*−*0.094	*−*1.812	0.008	0.164
major	*−*0.057	*−*1.108	*−*0.073	*−*1.403	0.080	1.676
R^2^	0.201		0.173		0.288	
F	15.946^***^		16.639^***^		32.290^***^	

*p* > 0.05, ^**^ *p* < 0.01, ^***^ *p* < 0.001.

**Figure 3:**
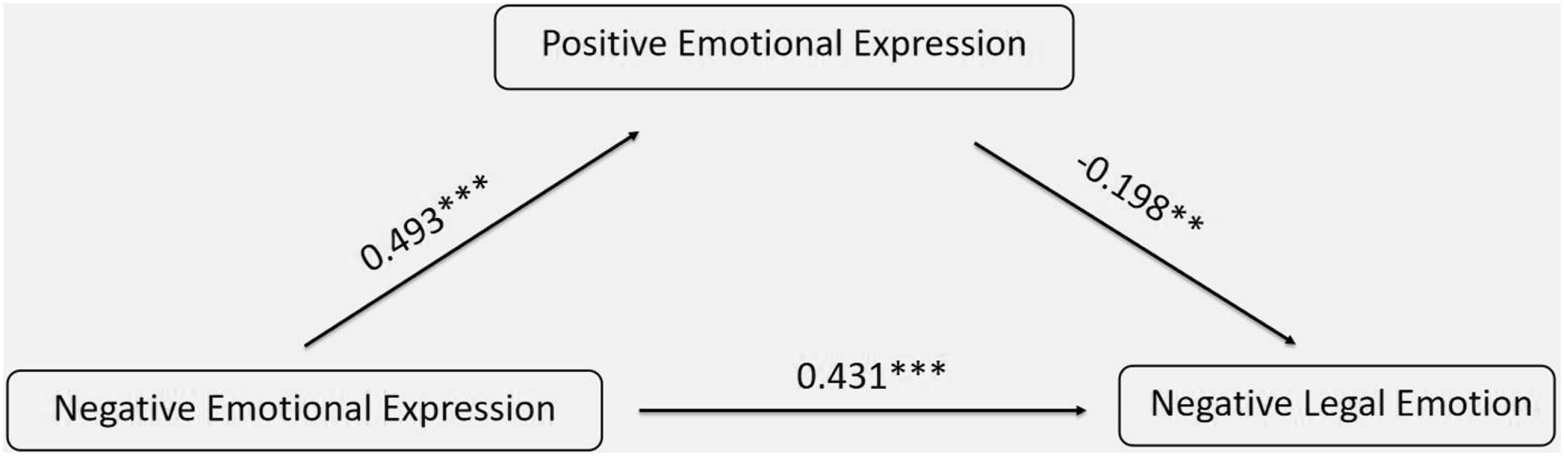
The Relationship between Negative Emotional Expression and Negative Legal Emotion: A Model Diagram of the Mediating Effect of Positive Emotional Expression.

The Bootstrap method was employed, with 5,000 repeated samples taken, to calculate 95% confidence intervals. The results indicate that the confidence intervals for each path tested did not include 0, indicating significant mediating effects. Among these, the mediating effect and the direct effect have opposite signs, suggesting the presence of a suppressor effect. For more details, please refer to Table 11. The model is shown in Figure 3.

**Table 11:** Mediation effects with bootstrapping.

	Effect	BootSE	BootLLCI	BootULCI
Indirect	*−*0.133	0.044	*−*0.228	*−*0.058
Direct	0.587	0.077	0.439	0.749
Total	0.454	0.076	0.306	0.605

### 2.3 Study 1 discussion

#### 2.3.1 Analysis of various factors affecting legal emotions

The correlation analysis found that there was a positive relationship between mother*-*children attachment and dependence, and a negative correlation between dependence and anxiety. In China, college students are still in their teenage years. The university stage is in the transition between school and society. Before the university stage, Chinese parents are more willing to take care of their children’s life and study, while their children are more eager to be independent, therefore, the anxiety of college students often comes from the transition of parental care. When a child comes to college, it is the first time he or she really leaves the family and goes to another city to study and live without parents, which is in a way the beginning of independence. At the same time, parents will also bear the tuition fees and living expenses in college. University life not only meets the students’ needs for freedom and independence, but also does not need to worry about the economic aspects of survival. Therefore, anxiety is negatively related to attachment.

The regression analysis in this study revealed that mother*-*children attachment positively predicts positive legal emotion among college students. According to attachment theory, secure mother*-*children attachment relationships are believed to provide individuals with a sense of security and emotional support, thereby facilitating the development of positive emotions (Ainsworth et al., 2015). When college students establish stable, intimate, and secure attachment relationships with their mothers, they are more likely to develop positive identification with and emotional experiences toward the law. As the primary attachment figure, mothers play a crucial role in meeting the emotional needs and satisfaction of college students. A positive mother*-*children attachment relationship can provide emotional support and guidance, leading to the formation of a positive emotional attitude towards the law. The predictive role of mother*-*children attachment in positive legal emotion may also involve processes of emotional transmission and modeling. As a significant role model, a mother’s attitudes and emotional expression can potentially influence college students’ legal emotions. If a mother holds a positive attitude towards the law and effectively expresses positive emotions, college students may develop similar legal emotions through imitation and learning. Positive emotional expression and attitudes within the mother*-*children attachment relationship can be transmitted to college students, thereby fostering the development of their positive legal emotions.

The mother*-*children attachment relationship can provide emotional support and regulation functions for college students. When facing legal issues, challenges, or uncertainties, a mother’s emotional support and guidance can assist college students in understanding and managing these emotions, enabling them to effectively cope with legal matters. The mother’s active involvement and support can enhance college students’ trust and identification with the law, thereby fostering the development of positive legal emotions.

Innovative spirit equips college students with the capacity and inclination to actively explore and engage with new knowledge and perspectives. The field of law is constantly evolving and developing, requiring continuous learning and understanding of new legal provisions, cases, and legal theories. College students with an innovative spirit are more inclined to proactively seek new knowledge. Their in*-*depth understanding and exploration of the law might lead them to develop positive legal emotions. Innovative spirit signifies that college students possess an open mindset and the ability to adapt to change. In the realm of law, different case scenarios may require students to exhibit flexible thinking and adaptability. College students with innovative spirit are better equipped to comprehend and address legal challenges, consequently nurturing positive legal emotions. Innovative spirit is closely related to the ability for creative problem*-*solving. In the field of law, college students may encounter various legal issues and challenges, necessitating the search for innovative and effective solutions. College students with innovative spirit are more inclined to think and explore diverse legal approaches. They are capable of developing creative solutions and, through the process of problem*-*solving, cultivating positive legal emotions towards law.

This study also found that positive emotional expression had a positive predictive effect on college students’ positive legal emotions. Analyzing the reasons, there could be several aspects to consider: based on the theory of the interaction between emotion and cognition, positive emotional expression can facilitate positive cognitive processes and conceptual formation (Clore and Huntsinger, 2007). When college students use positive emotional expression to convey their positive attitudes towards the law, they often tend to focus on and emphasize the positive aspects of the legal field in terms of cognition. This cognitive bias contributes to the formation of positive legal emotions, encouraging college students to be more willing to actively engage in legal activities and explore legal knowledge. The theories of self*-*motivation and achievement suggest that positive emotional expression can stimulate individuals’ self*-*motivation and sense of achievement (Goleman, 1996; Abe, 2011; Pekrun, 2014; Trigueros et al., 2019). When college students express their fondness and interest in law positively, they often feel more motivated and inclined to learn and engage in the field of law. This self*-*motivation and sense of achievement can further enhance their positive legal emotions, creating a virtuous cycle.

The study demonstrated that the dimensions of dependence and anxiety in adult attachment had a significant impact on college students’ negative legal emotions. The reasoning behind this could be that in intimate relationships, a person’s dependence on others reflects a tendency to seek support and comfort from them, leading to a positive attitude towards legal regulations that govern interpersonal relationships. Additionally, relying on others suggests a likelihood of having higher social support, and the law is also viewed as a form of social support when individuals face difficulties. Therefore, dependence can negatively predict college students’ negative legal emotions. The anxious dimension within adult attachment is often associated with feelings of insecurity and instability in intimate relationship models. This sense of insecurity may translate into mistrust and dissatisfaction with the legal system in the context of law. Anxious emotions can potentially lead individuals to have a cognitive bias towards negative and dangerous aspects. In the legal domain, anxious emotions might make individuals more prone to noticing and emphasizing the negative and challenging aspects of legal issues. This cognitive bias can reinforce the formation of negative legal emotions, causing individuals to be more inclined towards holding negative attitudes and emotions towards the law.

#### 2.3.2 Mechanism analysis of several influencing factors of legal emotions

The results of the chain mediation analysis in this study showed that the chain mediation effect of anxiety and dependency was significant in the influence of mother*-*children attachment on negative legal emotions. Based on the internal working models of attachment (Bowlby, 1982), an individual’s early attachment relationship with their mother influences their later relational state with others in intimate relationships. Therefore, individuals with a high degree of mother*-*children attachment tend to establish highly dependent intimate relationships with others, whereas individuals with a low degree of mother*-*children attachment may develop an anxious attachment style. Law is a social system that regulates the interests of individuals, and an individual’s relationship with the law can be seen as a manifestation of their social attachment to unspecified others. In this way, the attachment relationship with one’s mother not only affects an individual’s dependence or anxiety states in later intimate relationships but also has the potential to impact their broader relationships with others. Consequently, maternal attachment can influence not only an individual’s direct negative emotions towards the law but also, through adult attachment styles like dependency or anxiety, indirectly shape their overall negative (or positive) emotions towards the law. The observed chain mediation effect highlights the significant mechanism by which adult attachment plays a crucial role in mediating the relationship between maternal attachment and negative legal emotions among college students.

This study found that in the process of how entrepreneurial spirit influenced positive legal emotions among college students, positive emotional expression played a mediating role. Furthermore, in the process of how negative emotional expression affected negative legal emotions among college students, positive emotional expression also served as a mediator. Specifically, the innovative spirit may have influenced positive legal emotions among college students by inspiring positive emotional experiences, subsequently leading them to express positive emotions such as joy, optimism, and satisfaction. These positive emotional expressions could further enhance the students’ positive legal emotions. In the process of how negative emotional expression affected negative legal emotions, positive emotional expression played a mediating role. Specifically, negative emotional expression might trigger negative emotions, but through positive emotional expression, college students might seek emotional regulation and transformation towards positive emotions, thereby alleviating their negative legal emotions.

## 3 Study 2: the mechanism of legal emotion on aggressive behavior of college students

### 3.1 Study 2 method

#### 3.1.1 Sample

The participants of the study were university students from a college in Wenzhou, Zhejiang Province, China. The data collection was conducted by trained researchers in a group setting during classroom sessions. After eliminating invalid data, a total of 400 valid responses were retained. Among them, there were 182 male participants and 205 female participants. The distribution by academic year was as follows: 50 freshmen, 127 sophomores, 118 juniors, and 105 seniors. Furthermore, there were 201 participants from humanities and social sciences disciplines, and 199 participants from science and engineering disciplines. There were 129 participants whose fathers had an education level of junior high school or below, 148 participants with fathers having a high school or vocational school education, 112 participants with fathers having a bachelor’s or associate degree, and 11 participants with fathers having a master’s degree or above. As for mothers’ education levels, there were 166 participants with mothers having a junior high school education or below, 135 participants with mothers having a high school or vocational school education, 94 participants with mothers having a bachelor’s or associate degree, and 4 participants with mothers having a master’s degree or above.

#### 3.1.2 Procedures

The questionnaire response time for this study was approximately 20*-*25 min. Each participant was provided with informed consent, and to protect the privacy of the participants, the survey was conducted anonymously. This research obtained approval from the Ethics Committee of Wenzhou University.

#### 3.1.3 Measures

*The college student legal emotion scale* was developed by Xu (2020). This scale consists of two subscales: Positive Legal Emotion Scale and Negative Legal Emotion Scale. The scale consists of a total of 33 items. It utilizes a 5*-*point Likert scale, with scores ranging from 1 (“completely disagree”) to 5 (“completely agree”). A score of 1 corresponds to “completely disagree,” and a score of 5 corresponds to “completely agree.” Higher scores indicate higher levels of positive or negative legal emotions. The Cronbach’s α coefficients for the two subscales are 0.886 and 0.948, respectively. In the present study, the Cronbach’s α coefficients for positive legal emotion and negative legal emotion on this scale were 0.915 and 0.974, respectively.

*The Adolescent Social Alienation Scale (ASAS)* was developed by Yang et al. (2002). This scale comprises three major dimensions: social alienation, interpersonal alienation, and environmental alienation, with a total of 52 items. In this study, the focus is on the social alienation dimension, which includes a set of 22 items. The scale is assessed using a seven*-*point scale, where scores range from 1 (“completely disagree”) to 7 (“completely agree”). A score of 1 corresponds to “completely disagree,” and a score of 7 corresponds to “completely agree.” Higher total scores indicate a higher level of social alienation. In this study, the Cronbach’s α coefficient for this scale was 0.942.

*The Buss-Perry Aggression Questionnaire (BPAQ)* was developed by Buss and Perry (1992). The Chinese version of the Buss*-*Perry Aggression Questionnaire (BPAQ), revised by Liu et al. (2009), is used in this study. The scale consists of 20 items, organized into four subscales: Physical Aggression, Anger, Hostility, and Verbal Aggression. The scale employs a 5*-*point Likert rating system where “1” represents “extremely uncharacteristic of me” and “5” represents “extremely characteristic of me.” Higher scores indicate a higher level of aggression. In this study, the Cronbach’s α coefficient for the BPAQ was 0.948.

#### 3.1.4 Data analysis

The data processing and analysis were performed using SPSS 23.0 and the SPSS macro program PROCESS developed by Hayes (2013). The statistical analysis methods primarily involved correlation analysis and tests for mediation effects.

### 3.2 Study 2 results

#### 3.2.1 Test for common method bias

After the normal distribution test, the data conforms to a normal distribution. In this study, data collection was conducted using a questionnaire method. To mitigate common method bias, techniques such as setting reverse*-*scored items and ensuring anonymous responses were employed. After data compilation, Harman’s single*-*factor test was used for *post hoc* statistical control. The results of the test revealed the presence of 7 common factors with eigenvalues exceeding 1. The highest variance explained by the first factor was 37.53%, which is below the threshold of 40%. This indicates that there is no significant issue of common method bias in this study.

#### 3.2.2 Descriptive statistics and variable correlation analysis

As indicated in Table 12, the tendency towards aggressive behavior was negatively correlated with positive legal emotions (*r* = *−*0.270), positively correlated with negative legal emotions (*r* = 0.537), and positively correlated with social alienation (*r* = 0.760). Additionally, social alienation was negatively correlated with positive legal emotions (*r* = *−*0.313) and positively correlated with negative legal emotions (*r* = 0.510).

**Table 12:** Descriptive statistics and variable correlation analysis.

	M ± SD	Positive legal emotion	Negative legal emotion	Social alienation	Aggression
Positive legal emotion	47.540 ± 6.461	1			
Negative legal emotion	39.270 ± 19.341	*−*0.457^***^	1		
Social alienation	71.660 ± 26.106	*−*0.313^***^	0.510^***^	1	
Aggression	49.790 ± 17.949	*−*0.270^***^	0.537^***^	0.760^***^	1

***p < 0.001.

#### 3.2.3 Mediation model test of social alienation in the relationship between positive legal emotions and aggressive behavior

Using the PROCESS program developed by Hayes (2013), Model 4 was employed to test the mediating role of social alienation between positive legal emotions and aggressive behavior, while controlling for gender, grade, and major. The results, as presented in Table 13, indicated that positive legal emotions negatively predicted both aggressive behavior (*β* = *−*0.285, t = *−*5.876, *p* < 0.001) and social alienation (*β* = *−*0.320, *t* = *−*6.648, *p* < 0.001).

**Table 13:** The mediation effect of social alienation on the association between positive legal emotions and aggressive behavior.

Variables	Aggression		Social alienation		Aggression	
	*β*	*t*	*β*	*t*	*β*	*t*
Constant	94.019	12.145^***^	139.591	12.487^***^	22.646	3.667^***^
Positive legal emotion	*−*0.285	*−*5.876^***^	*−*0.320	*−*6.648^***^	*−*0.047	*−*1.358
Social alienation					0.744	21.729^***^
Gender	*−*0.084	*−*1.700	*−*0.069	*−*1.406	*−*0.033	*−*0.978
Grade	*−*0.082	*−*1.674	*−*0.014	*−*0.279	*−*0.072	*−*2.174^*^
Major	0.038	0.778	0.0008	0.016	0.038	1.135
R^2^	0.091		0.104		0.586	
F	9.822^***^		11.397^***^		111.662^***^	

***p < 0.01, *p < 0.05, *p* > 0.05.

When social alienation was introduced as a mediating variable into the model, the results revealed that social alienation significantly and positively predicted aggressive tendencies (*β* = 0.744, *t* = 21.729, *p* < 0.001). The influence of positive legal emotions on aggressive behavior was not statistically significant (*p* > 0.05), implying that social alienation fully mediated the relationship between positive legal emotions and aggressive behavior.

**Figure 4:**
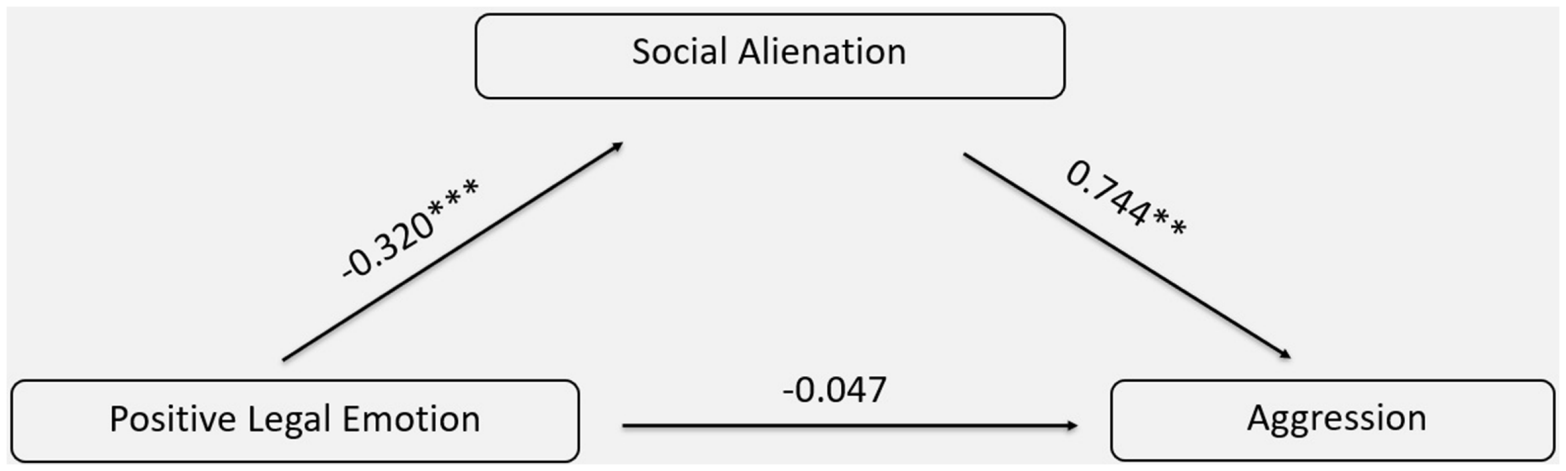
The Relationship between Positive Legal Emotion and Aggression: A Model Diagram of the Mediating Effect of Social Alienation.

The Bootstrap method was employed to perform 5,000 rounds of resampling for calculating 95% confidence intervals, and the outcomes were displayed in Table 14. The confidence interval for the total effect was [−1.056, −0.526], which did not encompass 0, indicating a significant total effect. The confidence interval for the direct effect was [*−*0.319, 0.058], which encompassed 0, signifying that the direct effect was not significant. The confidence interval for the indirect effect was [*−*0.936, *−*0.435], which did not include 0, suggesting a significant indirect effect. The proportion of the mediation effect was 83.57%. The model is shown in Figure 4.

**Table 14:** Mediation effects with bootstrapping.

	Effect	BootSE	BootLLCI	BootULCI	Proportion
Indirect	*−*0.661	0.126	*−*0.936	*−*0.435	83.57%
Direct	*−*0.13	0.096	*−*0.319	0.058	16.43%
Total	*−*0.791	0.135	*−*1.056	*−*0.526	

#### 3.2.4 Mediation model test of social alienation in the relationship between negative legal emotions and aggressive behavior

Utilizing the PROCESS program developed by Hayes (2013), Model 4 was employed to examine the mediating role of social alienation between negative legal emotions and aggressive behavior, while controlling for gender, grade, and major. The results, as depicted in Table 15, indicate the following: Negative legal emotions positively predicted both aggressive behavior (*β* = 0.539, *t* = 12.703, *p* < 0.001) and social alienation (*β* = 0.511, *t* = 11.730, *p* < 0.001).

**Table 15:** The mediation effect of social alienation on the association between negative legal emotion and aggressive behavior.

Variables	Aggression		Social alienation		Aggression	
	*β*	*t*	*β*	*t*	*β*	*t*
Constant	12.206	3.616^***^	45.486	7.109^***^	12.206	3.616^***^
Negative legal emotion	0.539	12.703^***^	0.511	11.730^***^	0.205	6.598^***^
Social alienation					0.655	18.014^***^
Gender	*−*0.013	*−*0.290	0.002	0.037	*−*0.014	*−*0.424
Grade	*−*0.086	*−*1.987^*^	*−*0.015	*−*0.331	*−*0.076	*−*2.380^*^
Major	0.044	1.008	0.0001	0.003	0.044	1.357
R^2^	0.298		0.261		0.615	
F	41.885^***^		34.822^***^		125.854^***^	

^***^p < 0.01, ^*^p < 0.05, *p* > 0.05.

Upon introducing social alienation as a mediating variable into the model, it was found that social alienation significantly and positively predicted aggressive behavior (*β* = 0.655, *t* = 18.014, *p* < 0.001). The positive predictive effect of negative legal emotions on aggressive behavior remained significant (*β* = 0.205, *t* = 6.598, *p* < 0.001), indicating that social alienation partially mediated the relationship between negative legal emotions and aggressive behavior.

**Figure 5:**
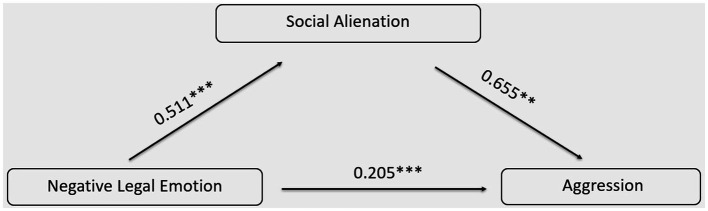
The Relationship between Negative Legal Emotion and Aggression: A Model Diagram of the Mediating Effect of Social Alienation.

Utilizing the Bootstrap method with 5,000 iterations, 95% confidence intervals were computed, and the results are presented in Table 16. The confidence interval for the total effect was [0.422, 0.578], which did not include 0, indicating a significant total effect. The confidence interval for the direct effect was [0.123, 0.256], also not encompassing 0, signifying a significant direct effect. The confidence interval for the indirect effect was [0.254, 0.376], which did not include 0, indicating a significant indirect effect. The proportion of the mediation effect was 62.00%. The model is shown in Figure 5.

**Table 16:** Mediation effects with bootstrapping.

	Effect	BootSE	BootLLCI	BootULCI	Proportion
Indirect	0.310	0.031	0.254	0.376	62.00%
Direct	0.190	0.034	0.123	0.256	38.00%
Total	0.500	0.039	0.422	0.578	

### 3.3 Study 2 discussions

Social alienation refers to an individual’s lack of engagement with social relationships and participation in society. It’s often associated with factors like social isolation, a lack of belonging, and inadequate social support. This sense of social alienation can lead individuals to develop indifferent or distant attitudes toward legal systems and social norms. Consequently, social alienation may exhibit a negative association with positive legal emotions and a positive association with negative legal emotions. In other words, stronger feelings of social alienation are linked to lower levels of positive legal emotions and higher levels of negative legal emotions.

Feelings of social alienation might lead individuals to experience isolation and helplessness, which can escalate feelings of fear and dissatisfaction within them. This negative psychological state could potentially trigger aggressive behavior—a tendency for individuals to resort to aggressive language or actions as a response to external pressures and challenges. Therefore, social alienation may mediate the relationship between legal emotions and aggressive behavior by augmenting the individual’s propensity towards aggression.

In this study, among college students, positive legal emotions could only exert an influence on aggressive behavior through the mediating role of social alienation. Having positive legal emotions reflects an individual’s degree of approval towards the legal system and its institutions, and it is also an expression of their acceptance within society. Therefore, enhanced positive legal emotions strengthen the individual’s connection with society, thereby reducing their sense of social alienation. Social alienation, in turn, signifies a disconnect between the individual and society, leading to feelings of isolation. Within such a social context, individuals may not experience a fundamental sense of belonging, prompting them to exhibit a greater tendency towards aggression as a way to cope with these setbacks.

Negative legal emotions among college students can influence aggressive behavior both through the mediation of social alienation and by directly affecting aggressive behavior. Negative legal emotions towards the legal system are also shaped through interpersonal interactions. If individuals experience unjust treatment in their social interactions or face unfair punishments within the legal system, they may develop a sense of disillusionment with others and society as a whole. Consequently, they might resort to aggressive behavior as a means to express their discontent with society. According to emotion regulation theory, when individuals undergo unfair treatment, they may resort to aggressive behavior as a direct expression of their discontent (Koole, 2009; Muhammad et al., 2023). In such cases, aggressive behavior serves as a means of emotional regulation. Therefore, negative legal emotions can directly predict aggressive behavior in a positive manner. This research outcome demonstrates that negative legal emotions towards the law can directly trigger an individual’s aggressive behavior. Therefore, addressing and alleviating negative legal emotional experiences among college students is a crucial approach to preventing the emergence of aggressive behavior.

Additionally, social alienation plays a pivotal role in the influence of legal emotions on aggressive behavior. Thus, enhancing the connection between individuals and society can further reduce the occurrence of aggressive behavior. This concept has been supported by Herschi’s crime theory and empirical studies. In this study, the perspective of legal socialization, such as fostering positive legal emotions within individuals, enhances their connection to society, ultimately leading to a reduction in aggressive behavior. This approach also stands as one of the strategies for preventing criminal behavior.

## 4 Comprehensive discussions

Through two sub*-*studies, this research investigated the influencing factors in the development of legal emotions among college students and further revealed the underlying mechanisms through which legal emotions affect aggressive behavior. Study 1 initially confirmed the impact of mother*-*children attachment on legal emotions among college students and revealed that mother*-*children attachment influences individuals’ attachment states with significant others, subsequently affecting their legal emotions. This micro*-*level perspective uncovers the mechanisms underlying the formation of individual legal emotions, with the theoretical foundation rooted in attachment’s internal working models. In Study 1, the introduction of attachment variables aimed to uncover the socialization process of legal emotions from a dynamic perspective, focusing on the process of legal emotion generation. On the other hand, the variables of emotional expression and innovative spirit emphasized individual factors of college students to explain the mechanisms underlying the influence of legal emotions. Therefore, Study 1 not only explored influencing factors from a dynamic occurrence perspective but also investigated the influence of legal emotions from a static individual trait standpoint. It revealed external factors in the formation of legal emotions and explained individual factors in the development of legal emotions. This sets the groundwork for the later intervention of legal emotions on aggressive behavior in Study 2.

Building upon the foundation of uncovering the factors influencing legal emotions, Study 2 endeavors to provide additional evidence regarding the impact of legal emotions on individual aggression. Its ultimate objective is to utilize legal emotions as a starting point for preventing the emergence of aggressive behavior, thus offering innovative perspectives for the prevention of juvenile delinquency. The empirical research of Study 2 confirmed that a significant variable influencing the impact of legal emotions on aggressive behavior is social alienation. These findings further corroborate the general theory of crime proposed by Hirschi as well as the theory of social control through law.

Therefore, after verifying that legal emotion does have an impact on aggressive behavior, a new perspective is provided for crime prevention, that is, from the perspective of individual legal socialization, such as cultivating individual positive legal emotion and dispelling individual negative legal emotion, so as to increase the connection between individuals and society, and finally achieve the goal of crime prevention. Cultivating individual positive legal emotions can be informed by the findings of Study 1. For instance, one approach could involve nurturing a healthy parent*-*child attachment relationship, with a particular focus on mother*-*child attachment, starting from an early age. Additionally, interventions aimed at promoting positive emotional expression and fostering an innovative mindset within individuals could also be employed to positively influence their legal emotions.

## 5 Conclusion

The present study, in Sub*-*study 1, revealed the influential roles of mother*-*children attachment and adult attachment in shaping the legal emotions of college students. Furthermore, it explored the impact mechanisms of innovation spirit and emotional expression on legal emotions from the perspective of individual factors among college students. Sub*-*study 2 further explored the topic by revealing that positive legal emotions only had an indirect impact on aggressive behavior through the mediating role of social alienation. Conversely, negative legal emotions not only directly affected aggressive behavior but also influenced it through social alienation. Therefore, social alienation played a mediating role in the impact of legal emotions on aggressive behavior.

## 6 Limitations and implications

This study explored the factors influencing legal emotions among college students through two sub*-*studies, as well as the mechanisms underlying the relationship between college students’ positive and negative legal emotions and their aggressive behavior. However, the research methodology employed in this study was a questionnaire*-*based survey. While it allowed for the exploration of explanatory factors for legal emotions and demonstrated the correlation between legal emotions and aggressive behavior, it could not establish a causal relationship between independent and dependent variables. Another limitation is that this study solely relied on self*-*report measures. Despite using methods like anonymity to enhance the authenticity of the data, responses to questions about attitudes or emotions towards current national laws could still be influenced by social desirability bias. Subsequent research could consider employing alternative methods, such as implicit testing methods like the Implicit Association Test (IAT).

The current study has some noteworthy strengths. Firstly, by investigating the influential factors of crucial legal emotions in legal socialization and their mechanisms in influencing aggressive behavior, this study has proposed a novel perspective for research on crime prevention. Secondly, the theoretical and empirical research on legal emotion has extended the scope of studies related to legal socialization. Thirdly, by incorporating both father*-*child and mother*-*child attachment into the same model as influencing factors of legal emotion, this study has analyzed their distinct explanatory power on positive and negative legal emotions.

## Data availability statement

The raw data supporting the conclusions of this article will be made available by the authors, without undue reservation.

## Author contributions

SX: Writing – review & editing, Writing – original draft, Visualization, Supervision, Resources, Project administration, Methodology, Investigation, Funding acquisition, Data curation, Conceptualization. JY: Writing – review & editing, Investigation, Data curation. LF: Writing – review & editing, Supervision. QY: Writing – review & editing, Supervision. ZW: Writing – review & editing, Supervision, Funding acquisition. YZ: Project administration, Writing – original draft.

## Ethics statement

The studies involving humans were approved by the Ethics Committee of Wenzhou University. The studies were conducted in accordance with the local legislation and institutional requirements. Written informed consent for participation in this study was provided by the participants' legal guardians/next of kin.
